# Non-thermal plasma accelerates the healing process of peripheral nerve crush injury in rats

**DOI:** 10.7150/ijms.44041

**Published:** 2020-04-27

**Authors:** Hyeong-Geun Lee, Jeong-Hae Choi, Yoon-Seo Jang, Uk-Kyu Kim, Gyoo-Cheon Kim, Dae-Seok Hwang

**Affiliations:** 1 Department of Oral & Maxillofacial Surgery, School of Dentistry, Pusan National University; 2 Department of Research and Development Center, FEAGLE Corporation, Yangsan, South Korea.; 3 Department of Oral Anatomy and Cell Biology, School of Dentistry, Pusan National University, Yangsan, South Korea

**Keywords:** Non-thermal plasma (NTP), Sciatic Nerve Crush Injury (SNCI), Skeletal Muscle Healing, Macrophage, Myelin Sheath, Neuronal Axon

## Abstract

The objective of this study was to evaluate the effect of non-thermal plasma (NTP) on the healing process of peripheral nerve crush injuries, which can occur during dental implant procedures. For this, a rat model of sciatic nerve crush injury (SNCI) was adopted. The rats were divided into three groups: non-nerve damage (non-ND), nerve damage (ND), and ND+NTP group. To evaluate the sciatic nerve (SN) function, the static sciatic index was calculated, and the muscle and SN tissues were subjected to a histologic analysis. The results showed that NTP effectively accelerated the healing process of SNCI in rats. In contrast to the ND group, which showed approximately 60% recovery in the SN function, the NTP-treated rats showed complete recovery. Histologically, the NTP treatments not only accelerated the muscle healing, but also reduced the edema-like phenotype of the damaged SN tissues. In the ND group, the SN tissues had an accumulation of CD68-positive macrophages, partially destroyed axonal fibers and myelinated Schwann cells. Conversely, in the ND+NTP group, the macrophage accumulation was reduced and an overall regeneration of the damaged axon fibers and the myelin sheath was accomplished. The results of this study indicate that NTP can be used for healing of injured peripheral nerves.

## Introduction

Peripheral nerve injuries (PNIs) are clinically encountered worldwide 1. Zhu at al reported an annual incidence rate of 0.1% 2. PNIs can incur approximately 150 billion dollar healthcare expense per year in the USA alone 3. Acute PNIs occur in 3~10% of the patients after a trauma to the extremities 4, mostly due to crush or penetrating injury and ischemia. Thermal injury, electric shock, radiation, and vibration injuries may also cause PNIs 5. The gold standard treatment for a severe nerve injury is surgical nerve repair including microsurgical nerve repair 6, nerve allografting 7, and tubularization techniques 8. Despite the recent improvements in the surgical treatment of PNIs, a complete recovery of the damaged nerve functions, particularly the motor functions, has not yet been achieved 9. Several non-surgical methods have also been suggested to enhance the healing process, 10 including the use of drugs such as vitamins B and E, dexamethasone, and alpha lipoic acid 11. However, the surgical treatment is invasive, and pharmacotherapy alone is not effective to restore the nerve function. Therefore, newer therapeutic methods for the treatment of peripheral nerve damage are needed.

Non-thermal plasma (NTP) is a useful medical technique with several biological functions 12. In physics, the term “plasma” refers to an ionized gas 13. During the generation of NTP, several working elements, such as the reactive oxygen and nitrogen species, other chemicals, electronic fields, and electrons can be generated 14. These working elements are expected to regulate several biological reactions 15. Thus far, strong anti-bacterial 16-18 and anti-inflammatory 19, 20 activities of NTP have been reported, and a possible role of NTP in treating cancer has also been suggested 21, 22. In dentistry, NTP has not only been utilized as a bactericidal agent 17, but also for tooth whitening 23, 24 and preservation 25. However, the most promising medicinal property of NTP is the acceleration of wound healing 14. Depending on the methods used to generate NTP, its topical application can promote faster wound healing by minimizing bacterial infection 26, 27 and accelerating tissue regeneration 28, 29. Despite the beneficial action of NTP on cutaneous wounds, its effects on subdermal wounds, particularly on the physically damaged nerves, have not been reported.

In this study, the effect of NTP on the functional recovery of the damaged sciatic nerve was analyzed using a rat model of sciatic nerve crush injury (SNCI). The rat behavior was monitored and analyzed using the static sciatic index (SSI). To evaluate the effects of NTP on the damaged muscles and sciatic nerve (SN) tissues, histological and immunofluorescence (IF) analyses were performed. The study aimed to assess the possible role of NTP in the PNI healing process.

## Methods

### NTP device

**Figure 1:**
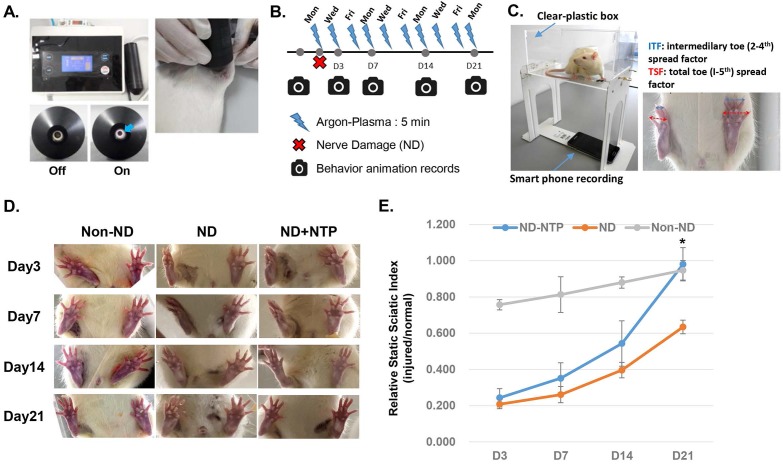
** NTP fastened the recovery of the SN function**. (A) Photographs showing the NTP device used in this study and the methods for the animal treatment. This device ejected argon-based coaxial-DBD plasma, and during the 5 min of NTP treatment, the distances between the NTP ejecting module and the sutured skin was kept at 5 mm using a spacer. (B) A schematic diagram describing the animal experiment schedule. (C) A photograph explaining the methods for the video recording of animal feet movements using a smart phone. (D) Representative images showing the SN function at 3, 7, 14, and 21 days after the SNCI operation. (E) The statistical results showing the effect of NTP on the recovery of the SN function. Data shown are representative of each group (n=6), * p<0.05.

For this study, an NTP-generating device developed by Feagle Corporation (Yansan-si, Gyeonsangnam-do, South Korea) was used (Fig. 1a). This device has a coaxial dielectric barrier discharge (DBD)-type plasma jet generating module which consists of two electrodes and one dielectric: an inner electrode made of stainless steel and a 10 mm wide-area of alumina tube, with a relative permittivity of 9.1, wrapped with copper tape as an outer electrode. The outer electrode was connected to a sinusoidal high-voltage circuit generating a peak-to-peak voltage of 3 kV with a frequency of 15 kHz. The inner electrode was grounded to prevent the transition from the glow discharge to the arc discharge between the inner electrode and the skin of the rats. The working gas was argon, which flowed between the inner electrode and alumina tube at a flow rate of 2 slm (standard liters per minute). Plasma generated in this device did not extend outwards like a plasma jet but was generated between the inner and outer electrodes. The temperature of the NTP flow at the end of the electrode was maintained at less than 35 °C for 10 min. The ultraviolet (UV) sensors did not detect any UV radiation from the device, however, the generated plasma predominantly consisted of OH radicals. The detailed chemical properties of the plasma from this device can be found in our previous report 29.

### The rat SNCI model setup and NTP treatment

The SNCI model of Varejão et al. was adopted and slightly modified 30. A total of 18 male Wistar A rats (8 weeks old, weighing approximately 250 g) were procured from Samtaco Corporation (Osan-si, Gyeonggi-do, South Korea) and used for this study. The rats were equally divided into three groups (n=6): non-nerve damage (non-ND), nerve damage (ND), and ND+NTP. In the non-ND group, the SN was exposed surgically but without the crush injury procedure. The ND group was a negative control group in which the rats underwent the SNCI procedure, but the healing was dependent only on the natural healing process. The ND+NTP group was an experimental group in which the rats were treated with NTP after the SNCI procedure. For the surgical operation, all the rats were anesthetized with an intraperitoneal injection of a ketamine/xylazine cocktail at a concentration of 100 mg/kg ketamine (Yuhan, Seoul, South Korea) and 10 mg/kg xylazine (Rompun, Bayer Korea Ltd, Seoul, South Korea). A subcutaneous injection of 0.3 mL of lidocaine solution was used to induce local anesthesia (Lidocaine HCl 2% Injection, Huons, Seongnam, South Korea). For surgical antibiotic prophylaxis, injection cefazolin (50 mg/kg) was administered. The hind legs of the anesthetized rats were shaved and disinfected with betadine (povidone-iodine). The skin was incised from the hip joint parallel to the femur, and the thigh muscle layer was carefully dissected and incised. The SN was observed clearly, and the sciatic trifurcation was exposed. In the ND and ND+NTP groups, the SN was compressed for 60 s using a microvascular clamp (for 2.0-5.0-mm vessels, S&T, Neuhausen, Switzerland) and Kelly forceps to produce the SNCI. The width of the microvascular clamp at the nerve compression area was maintained at 2.5 mm, and the SNCI was induced at a point 5 mm proximal to the sciatic trifurcation. For the non-ND group, the same point of SN was surgically exposed as in the other groups, but it was immediately set back without any physical damage. After the surgical procedure, the muscle layers at the surgical site were sutured with synthetic absorbable sterile sutures (coated polyglactin 910 (Vicryl), 4-0 size) using the simple interrupted suturing technique, and the incised skin layer was sutured with synthetic non-absorbable monofilament sutures (nylon (Ethilon), 4-0 size) using the continuous suturing technique. The NTP treatment in the ND+NTP group was performed immediately after the SNCI procedure. NTP was applied topically for 5 min at a 5 mm distance from the sutured skin (Fig. 1a). For the second NTP treatment and thereafter, the rats in all three groups were anesthetized by an intraperitoneal injection of a ketamine/xylazine cocktail, but only those in the ND+NTP group received the NTP treatment. The treatment was performed on the same 3 days every week (Monday, Wednesday, and Friday) for 3 weeks (Fig. 1b).

### Study approval

All experimental protocols for the animal experiments were reviewed and approved by the Animal Ethics Committee of Pusan National University (ED-PNU2017-0183), and all the animal procedures were performed in accordance with the relevant guidelines.

### Rat foot movement recording and SSI calculation

The rat behavior was recorded prior to the surgery and just before the NTP treatment on Days 3, 7, 14, and 21. A specially produced box made of rectangular transparent acrylic plates and measuring 25×16×12 cm was used (Fig. 1C). Video recording was done on a cellular phone with an in-built camera (i-Phone 6S Plus, Apple, CA, USA). To determine whether the SNCI operation (which was performed on a Wednesday) caused similar defects in the ND and ND+NTP groups, behavioral videos were recorded on Day 3 (Friday) and then every Wednesday thereafter. The rats were allowed to move freely within the transparent box, and videos were recorded for a duration of 1 minute.

For analyzing the SN function defects, the SSI was calculated using the static video method proposed by Bervar 31, as follows:

Static Sciatic Index (SSI) formula:

SSI = 108.44×TSF + 31.85×ITF - 5.49

TS: total toe spread, 1st-5th toe spread distance (mm)

IT: intermediary toe spread, 2nd-4th toe spread distance (mm)

The "F" of this equation is a consistent value, F=563.357

The SSI values of the right and left hind legs were measured, and the relative SSI values were calculated to assess the recovery rate of SN function after the SNCI operation.

### Histological analysis

One day after the final NTP treatment, all the rats were sacrificed, and the muscle and nerve tissue of the surgical area were isolated. All the tissue samples were fixed with 4% paraformaldehyde for 24 h, and then embedded in paraffin. The tissue sections (5 μm) were subjected to hematoxylin and eosin (H&E) staining, and the muscle and nerve tissue were then visualized under light microscopy (CX31, Olympus, Tokyo, Japan). Images were captured using an iCM 9.0 digital camera system (IMT I-solution Inc., NY, USA).

### IF analysis

Tissue sections (5 μm thick) were treated with antibodies against CD68 and myelin basic protein (MBP) (Santa-Cruz Biotechnology, Santa Cruz, CA, USA), neurofilament 200 (NF-200) and S100 (Abcam, Cambridge, MA, USA), and type 1 A collagen (Novous Biology, Centennial, CO, USA), and incubated for 2 h at 37℃. After being washed four times with phosphate buffered saline, the tissue sections were treated with anti-mouse Alexa Fluor-488 and anti-rabbit Alexa Fluor-594 (Thermo Fisher Scientific, Rockford, IL, USA) for 1 h at 37℃. After washing as above, the nuclei of the cells within the tissues were counterstained with 4',6-diamidino-2-phenylindole. The fluorescence from the tissues was observed, and images were captured using a Carl Zeiss LSM 780 confocal laser microscope.

### Data analysis

Data are presented as means ± standard errors of the means (SEM) of five independent experiments. The two-tailed Student's *t*-test was used to assess the differences in the mean values, and the level of significance was set at p < 0.05.

## Results

### Topical application of NTP accelerated the recovery of sciatic nerve function after the crush injury

The effectiveness of the wound surface treatment using NTP on the recovery of SN injury, was investigated by the behavioral analysis of the rats. The non-ND group showed minor defects in the SN function (approximately 20% decrease compared with the non-operated left hind foot) at 3 days after the surgery (Figs. 1C and D). Conversely, the rats in the ND and ND+NTP groups could not spread their toes, and showed an approximately 78-80% decrease in the SN function. Although after the third (Day 7) and the sixth (Day 14) NTP treatment, no statistically significant changes were seen between the ND and the ND+NTP group, the overall healing tendency of the latter was much faster. Moreover, the SN function of the NTP-treated rats was almost completely restored after nine treatments (Day 21), whereas that of the ND group recovered only up to 61%, indicating a significant difference.

### NTP increased the muscle fiber density and reduced the inflammatory reactions in the sutured area

**Figure 2:**
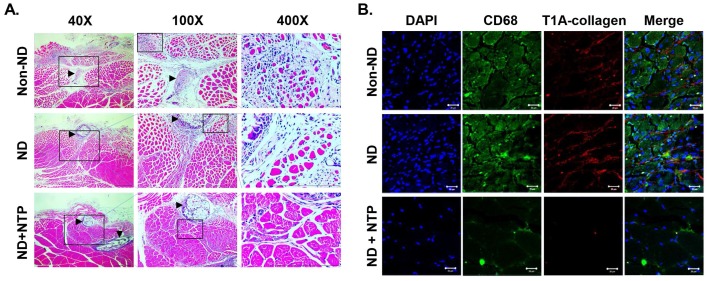
** NTP accelerated the healing process of the damaged skeletal muscle.** (A) The results of H&E staining of skeletal muscles that were damaged during the SNCI operation. Data shown are representative of each group (n=6), scale bar: 100 μm. (Arrowhead: suture region) (B) The expression of CD68 and Type I A collagen in the damaged skeletal muscles was visualized by IF coupled with confocal microscopy. DAPI was used for nuclear staining. Data shown are representative of each group (n=6), scale bar: 20 μm.

The effect of the NTP treatment on the recovery of injured muscle tissue covering the nerve tissue was examined prior to the direct examination of NTP's effects on the nerve tissue. On H&E staining (Fig. 2A), the naturally healed muscle tissues in the non-ND and ND groups exhibited finely divided myofibers (stained in red) in the sutured area (arrow head). These muscle tissues also had wide empty spaces between the myofibers. Conversely, the muscle tissues in the ND+NTP group had an increased size and density of myofibers around the sutured area. To further confirm the effect of NTP on inflammatory reactions, which delay the muscle healing process, and its effect on muscular fibrosis, an IF assay against the macrophage marker CD68 protein and type I collagen was performed. The muscle tissues in the ND+NTP group had significantly reduced number of nuclei and type I collagen expression than that in the non-ND and ND groups (Fig. 2B). Interestingly, CD68-expressing cells were abundant in the non-ND and ND groups but were reduced in the ND+NTP group.

### NTP accelerated the recovery of the damaged sciatic nerve tissue

**Figure 3:**
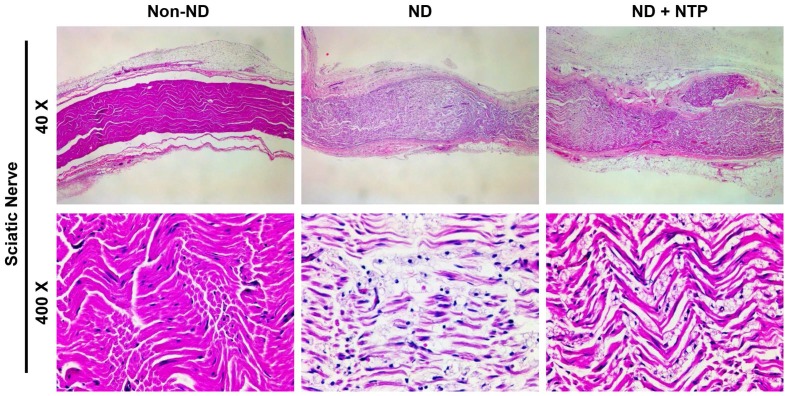
** NTP reduced the SNCI mediated edema-like phenotypes in the SN tissues.** SN tissues were subjected to H&E staining, and the photographs were taken using optical microscopy at 40X and 400X magnification. Data shown are representative of each group (n=6).

To examine whether the NTP-induced recovery of the SN function was accomplished through the recovery of damaged nerve tissue, the SN tissues were stained with H&E staining. As shown in the 40× magnification image of the stained tissue (Fig. 3), the SN of the ND group was thicker than that of the non-ND group, and a considerable empty space was observed between the SN fibers. Nine NTP treatments failed to reduce the thickness of the SN tissue to normal levels, but they increased the density of the SN fibers in the ND+NTP group as compared those in the ND group. This phenomenon was better appreciated in the higher magnification (400×) images. The SN in the ND group had short pinkish fibers with a lower density than that in the non-ND group, while that of the ND+NTP group had long well-connected fibers with a higher density than that in the ND group.

### NTP treatment helped the axonal and myelin sheath regeneration after the SNCI

**Figure 4:**
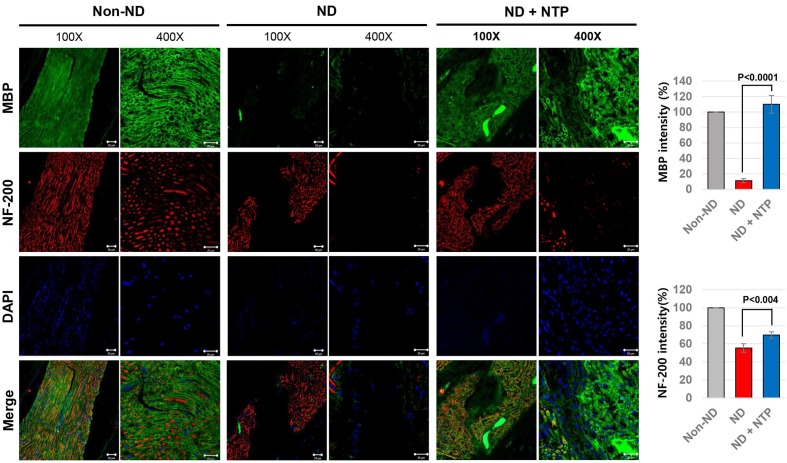
** NTP stimulated the axonal regrowth and myelin sheath formation in the damaged SN.** SN tissues were subjected to the IF assay using anti- NF-200 and MBP antibodies to show the recovery of neuronal axon and myelin sheath. The representative photographs of each group (n=6) were taken using confocal microscopy, scale bar: 20 μm.

To confirm the functional recovery of the damaged SNs at the tissue level, the SN tissues were subjected to IF staining against neurofilament heavy chain (NF-200) protein and MBP as markers of axon filament and myelinated Schwann cells, respectively 32. The results showed that the axon filament disconnection occurred in the SN tissues of the ND group, whereas the formation of the bridge filament, connecting the disconnected axon filaments, was observed in the ND+NTP group (Fig. 4). No recovery of the damaged myelin sheath was observed in the ND group, while the SN tissues of the ND+NTP group had higher amounts of MBP, indicating an active recovery of the myelin sheath.

### NTP accelerated the myelin sheath formation after the SNCI by reducing CD68 positive macrophage accumulation

**Figure 5:**
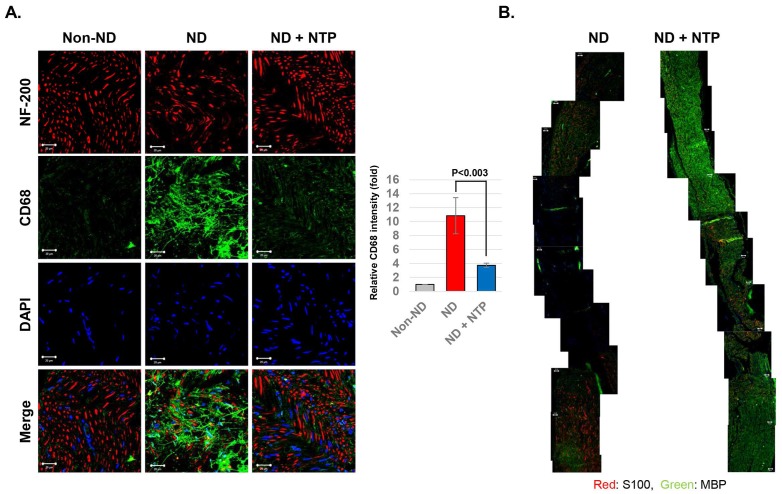
** NTP-induced myelin sheath recovery by decreasing CD68 positive macrophages within the damaged SN tissues.** (A) The axons (NF-200) and inflammatory macrophages (CD68) in SN tissues were visualized by confocal microscopy. Data shown are representative of each group (n=6), scale bar: 20 μm. (B) The myelinated (MBP) and non-myelinated (S100) Schwann cells in the SN tissues were visualized by merging the photographs of the confocal microscopic observation at 100 X magnification. Data shown are representative of each group (n=6), scale bar: 20 μm.

To determine the effect of NTP on inflammatory macrophages after the SNCI, the SN tissues were subjected to IF staining using antibodies against CD68 and NF-200 proteins. The SN tissues of the ND+NTP group showed a lesser accumulation of CD68 positive macrophages and no dendritic cells. In contrast, the ND group had an increased number of dendritic cells and CD68 positive macrophages near the neurofilament (Fig. 5A), indicating that the macrophage-mediated destruction of the damaged nerve was still incomplete.

To investigate the effect of NTP on Schwann cell proliferation and myelin sheath formation during the recovery after the SNCI, IF staining was performed using the antibodies against the non-myelinated and myelinated Schwan cell markers - S100 and MBP, respectively. In the SN tissues of the ND group, both types of Schwann cells were absent in the area around the crush injury (Fig. 5B). Conversely, in the ND+NTP group, non-myelinated Schwann cells were observed intermittently, while myelinated Schwann cells were observed in most regions.

## Discussion

Acceleration of wound healing is the most well-known medicinal property of NTP, but most studies have focused on cutaneous wound healing. The application of NTP has been reported for healing various types of wounds, such as regular cutaneous wounds, burn wounds, and wounds in animal models for diseases in which the natural healing is difficult. However, the effect of NTP on the damaged subdermal tissues or organs was rarely reported and its role in the healing of traumatic PNIs has not been evaluated.

In this study, the effect of NTP on the recovery of damaged peripheral nerves located under the skin and muscle tissues was examined. The well-established rat SNCI model 30, 33, 34 was adopted, and the effect of NTP on the recovery of the damaged SN tissues was investigated. The results of the SN function analysis showed that the SNCI procedure reduced the SN function by approximately 80% (ND and ND+NTP groups) at 3 days after the operation, whereas the simulated operation (non-ND group) reduced the SN function by only 20% (Fig. 1E). This means that 20% of the 80% relative SSI changes observed after the SNCI procedure could be attributed to the skin- and muscle-tissue damage and the remaining 60% to the destruction of the SN tissue. Interestingly, in contrast to the rats of the non-ND and ND group who showed a 60% recovery rate, the SN function was fully recovered in the rats of the ND+NTP group. These data indicate that the NTP treatment accelerated the healing process of the SN.

Since NTP was applied to the surface of the sutured muscle and skin tissues after the SNCI, the possibility of NTP affecting the recovery of the muscle tissue was examined histologically. The muscle tissues of the non-ND and ND groups showed a marked decrease in muscle tissue density near the sutures, and mono-nuclear cells were also significantly accumulated in the lesion (Fig. 2B). Conversely, in the ND+NTP group, the diameter of the muscle fibers was increased, and the number of mono-nuclear cells in the space between the muscle fibers was significantly decreased. Choi et al. reported that the nitrogen gas-based NTP treatment promoted muscle wound healing by promoting satellite cell proliferation and differentiation during the cutaneous wound regeneration in a subcutaneous fascia wound model in rats 35. Therefore, the increased muscle fiber density caused by the NTP in this study might also have been caused by the NTP-mediated satellite cell activation, although these cells were not directly subjected to the treatment.

The most common symptom of the natural healing process of the injured muscles is the increased number of the macrophages and fibroblasts between the muscle fibers, which induce the accumulation of collagen. When the muscles are restored normally, the amount of collagen decreases, but in chronic muscle wounds, the amount of collagen remains higher 36. The results of IF staining for CD68 and type I collagen performed in this study revealed that the amount of type I collagen was significantly higher in the non-ND and ND groups than in the ND+NTP group. Furthermore, the treatment significantly reduced the amount of macrophages in the muscle tissues with sutures (Fig. 3B). Therefore, NTP could be used to prevent the occurrence of chronic muscle wounds as it effectively minimized the inflammatory reactions mediated by the macrophages, and thereby blocked the accumulation of collagen in the wounded muscles.

In this study, the NTP treatment of the skin surface also promoted the healing of SN tissues located deeper in the muscle tissues. The results of H&E staining of the SN tissues revealed that the rats in the ND group had an edema-like phenotype and empty spaces between the short fibers. Conversely, the NTP-treated rats had dense and longer fibers than those in the ND group (Fig. 3). The effect of NTP on the damaged SN tissues was better appreciated in the results of specific staining for the myelin sheath and axons. MBP was not detected and some partially destroyed axon fibers remained in the SN tissues of the naturally healed rats in the ND group. Conversely, in the SN tissues of the ND+NTP group, the myelin sheath was restored in almost all the areas and some of the disconnected axon fibers were linked by bridge-like axon fibers (Fig. 4) These results histologically explained the effect of the NTP treatment on the accelerated recovery of the SN function (Fig. 1).

In the healing process of the PNIs, the damaged Schwann cells secrete various pro-inflammatory chemokines which invoke the inflammatory macrophages around the injured nerves. These macrophages perform a specific process called the “Wallerian degeneration” (WD), involving the removal of the damaged axons and myelin sheath 37. Recovery of damaged Schwann cells and axons occurs following the WD, and the role of the macrophages must be precisely controlled for the successful recovery of the PNIs. In this study, the repeated NTP treatment effectively reduced the amount of inflammatory macrophages in the SN tissues damaged by the SNCI procedure. In contrast to the SN tissues of the ND group, which had increased CD68-positive dendritic macrophages even at 3 weeks after the SNCI procedure, the SN tissues of the ND+NTP group showed lower CD68 protein expression (Fig. 5a). This may be directly related to the results of staining for the myelinated (MBP) and non-myelinated Schwann cell markers (S100), both of which were scarce in the vicinity of the SNCI site in the ND group (Fig. 5b). Conversely, the SN tissues in the ND+NTP group had completely recovered MBP-positive myelin sheath in all the regions. These results indicate that in the NTP-treated group, WD was suppressed and thus the myelin sheath formation and axon regeneration occurred over the entire region, while in the SN tissues of the ND group, the WD continued. In our previous studies, the potent anti-inflammatory activity of an argon plasma device similar to the NTP device used in this study was elucidated using an animal model of atopic dermatitis 19. Although the devices and the study animals were different, the present study also suggested that the anti-inflammatory activity of the NTP might shorten the WD process by blocking the inflammatory macrophages.

Overall, this study showed that the wound healing efficacy of NTP can be effectively applied to the SNCI. In addition to the functional recovery of the SN, NTP was also effective in restoring the muscle injuries incurred during the surgical procedures, suggesting the possibility that NTP could be used in the aftercare of various surgical procedures. Thus, we recommend NTP as a new tool for treating PNIs.

## Abbreviations

NTP: non-thermal plasma
SNCI: sciatic nerve crush injury
ND: nerve damage
SN: sciatic nerve
SSI: static sciatic index
PNI: peripheral nerve injury
slm: standard litter per minute
DBD: dielectric barrier discharge
H&E: hematoxylin and eosin
NF-200: neurofilament heavy chain
MBP: myelin basic protein
WD: Wallerian degeneration
